# The DIANA-mirExTra Web Server: From Gene Expression Data to MicroRNA Function

**DOI:** 10.1371/journal.pone.0009171

**Published:** 2010-02-11

**Authors:** Panagiotis Alexiou, Manolis Maragkakis, Giorgio L. Papadopoulos, Victor A. Simmosis, Lin Zhang, Artemis G. Hatzigeorgiou

**Affiliations:** 1 Biomedical Sciences Research Center “Alexander Fleming”, Institute of Molecular Oncology, Varkiza, Greece; 2 School of Biology, Aristotle University of Thessaloniki, Thessaloniki, Greece; 3 Institute of Computer Science, Martin Luther University Halle-Wittenberg, Halle, Germany; 4 Ovarian Cancer Research Center, University of Pennsylvania, Philadelphia, Pennsylvania, United States of America; 5 Computer and Information Sciences, University of Pennsylvania, Philadelphia, Pennsylvania, United States of America; Tel Aviv University, Israel

## Abstract

**Background:**

High-throughput gene expression experiments are widely used to identify the role of genes involved in biological conditions of interest. MicroRNAs (miRNA) are regulatory molecules that have been functionally associated with several developmental programs and their deregulation with diverse diseases including cancer.

***Methodology/Principal Findings*:**

Although miRNA expression levels may not be routinely measured in high-throughput experiments, a possible involvement of miRNAs in the deregulation of gene expression can be computationally predicted and quantified through analysis of overrepresented motifs in the deregulated genes 3′ untranslated region (3′UTR) sequences. Here, we introduce a user-friendly web-server, DIANA-mirExTra (www.microrna.gr/mirextra) that allows the comparison of frequencies of miRNA associated motifs between sets of genes that can lead to the identification of miRNAs responsible for the deregulation of large numbers of genes. To this end, we have investigated different approaches and measures, and have practically implemented them on experimental data.

***Conclusions/Significance*:**

On several datasets of miRNA overexpression and repression experiments, our proposed approaches have successfully identified the deregulated miRNA. Beyond the prediction of miRNAs responsible for the deregulation of transcripts, the web-server provides extensive links to DIANA-mirPath, a functional analysis tool incorporating miRNA targets in biological pathways. Additionally, in case information about miRNA expression changes is provided, the results can be filtered to display the analysis for miRNAs of interest only.

## Introduction

MicroRNAs (miRNA) are short, approximately 22 nucleotides long, endogenously expressed RNA molecules that regulate gene expression by binding, in a sequence specific manner, to the 3′ UnTranslated Region (3′UTR) of messenger RNA (mRNA) molecules [1]. MiRNAs are not only present but can also be abundant in eukaryotic cells, controlling a wide variety of target genes [2]. In the past few years, miRNAs have been associated to the regulation of a wide range of biological processes [3].

High-throughput methods for gene expression profiling are being massively used in recent years. Such methods strive to describe specific transcriptomic states of a cell and can identify changes in expression levels between cell states of interest. Since miRNAs often regulate large numbers of mRNAs [4], there are cases where deregulated miRNAs are responsible for a large part of gene expression changes. MicroRNA expression levels may or may not be experimentally measured in such experiments. However even if miRNAs that are down- or upregulated are known, there is always the possibility that only a subgroup of those miRNAs would be responsible for the changes in the transcriptome.

**Figure 1:**
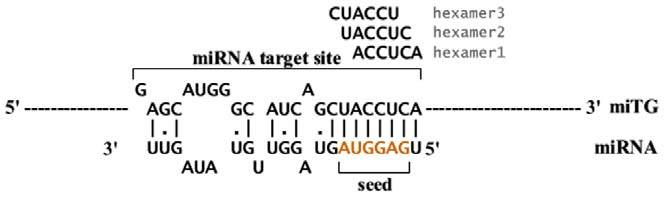
A miRNA molecule binds to a miRNA target gene (miTG). Hexamers 1,2 and 3 correspond to six nucleotide long sequences on the 3′UTR complementary to the first nucleotides of the miRNA . Hexamer 2 is the sequence complementary to the ‘seed’ of the miRNA, which has been suggested as the most important region for miRNA:miTG binding.

Such miRNAs may be identified via computational analysis, based on the fact that miRNAs target mRNA transcripts in a sequence dependent manner (Figure 1). Although it is known that miRNAs usually bind to specific sites in the 3′UTR region of targeted mRNA transcripts, the accurate identification of all miRNA target genes has not been possible yet. MiRNA binding sequences often tend to be overrepresented in sets of miRNA regulated genes compared to a random selection of genes [4], [5]. Different methods have been previously used to identify over- or under- expressed miRNAs through changes in the levels of their target genes. Essentially, the procedure followed by all such approaches is to identify differentially expressed genes, identify motifs that are overrepresented in these genes and then connect these motifs back to miRNAs. In an analysis performed by Lim et al [4] a motif discovery tool, MEME (Multiple Em for Motif Elicitation)[6], was used in order to identify motifs of six or more nucleotides in length that were significantly overrepresented in 3′UTR sequences of genes downregulated after hsa-miR-1 overexpression, compared to random 3′UTR sequences. The hexamer corresponding to position 2–7 of hsa-miR-1 was identified as the most significantly overrepresented motif.

In a similar experiment, Krutzfeld and colleagues [5] investigated the role of miRNA mmu-miR-122a in gene expression by neutralizing the miRNA through antagomirs and measuring the gene expression in wild type and knockdown cells. In a more sophisticated approach, they used the Wilcoxon Rank Sum test to compare hexamer frequencies between deregulated and unchanged genes between the two conditions. This analysis revealed that the frequency of the motif corresponding to the seed of mmu-miR-122 was significantly overrepresented in the 3′UTRs of upregulated genes and underrepresented in the 3′UTRs of downregulated genes.

Following this discovery, two freely available programs have been developed that perform similar computational analyses. MiReduce [7], [8], uses the correlation of the genome wide mRNA log fold changes of genes against the motif content of their 3′UTRs. Each motif contained in the 3′UTR contributes linearly to the fold change prediction. The method iteratively calculates which motifs contribute most to the level of change of genes. Sylamer [9] is another software package that identifies overrepresented occurrences of sequences in a ranked list of genes using the hypergeometric p-value distribution. This approach calculates frequencies for hexamers 1, 2 and 3 as well as 7mers (positions 1–7, 2–8) and 8mers (positions 1–8, 2–9) and involves corrections for nucleotide biases. The p-values of each motif are compared to all other motifs. From the user point both programs have to be downloaded and compiled and include a limited data format as input. MiReduce outputs text files whereas Sylamer includes a java based graphical interface.

Given the broad impact of miRNAs in different development stages and diseases we have felt the emerging need for a tool that provides such investigations in a fast and user-friendly way. We believe that it is imperative that such a resource be platform independent and easy to use. A web-based implementation seems as the obvious choice. In this light, we have developed DIANA-mirExTra, an interactive and fully web based application that can be easily used by non-experts. Besides a motif analysis, the web server offers the option to use evolutionary information in order to refine results. Additionally, it allows the use of different nomenclatures for gene names as input and provides direct links to miRNA target prediction and functional analysis applications.

## Results

**Figure 2:**
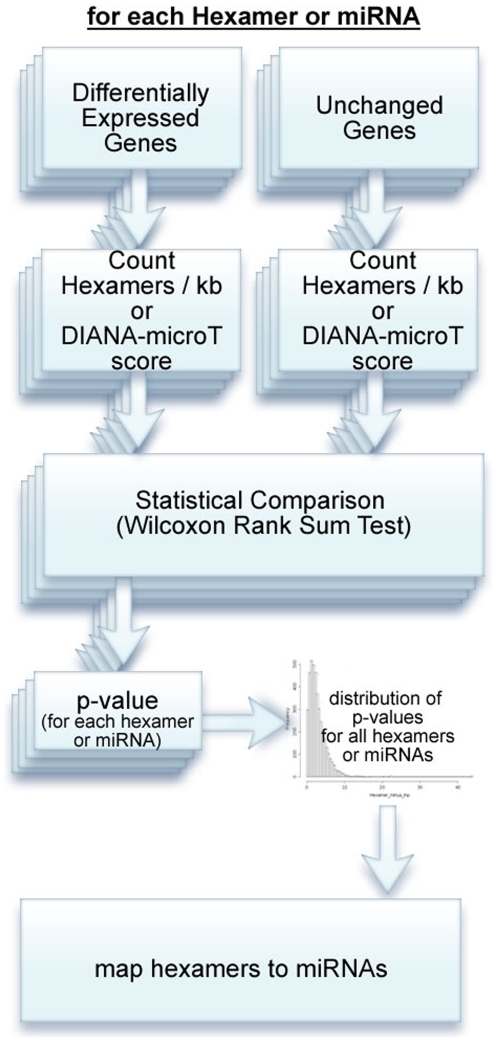
Overview of the algorithm. For each possible hexamer, the occurrences on the 3′UTRs of changed and unchanged genes are counted. The counts are compared using the Wilcoxon Rank Sum Test and a p-value produced. The distribution of p-values is plotted in a histogram. Hexamers are mapped back to known miRNA sequences (see Figure 1). When DIANA-microT target prediction scores are used, the Wilcoxon Rank Sum Test is performed between scores of changed and unchanged genes. A p-value is calculated for each miRNA and a corresponding histogram is produced. The histogram and sorted p-values are returned to the user in the Results page (see Figure 4).

The basic analysis flow of DIANA-mirExTra (www.microrna.gr/mirextra) is outlined in Figure 2. In the following section we will discuss each step of the algorithm in detail

### Input Data

The input to the web-server is two sets of genes (changed and unchanged genes). The user is given the options to use a form in the webpage or to upload a file with the relative gene names. Gene names can be provided in any of a wide range of commonly used nomenclatures (Ensemble gene and transcript IDs, RefSeq IDs, HUGO, Affymetrix probe codes) and are automatically translated to Ensemble Gene IDs. The Ensemble database is the base for the sequences and gene names used by the program. The first list contains genes whose expression levels have been found to be significantly changed in a high-throughput experiment. The second list consists of background genes, which are usually genes that did not significantly change their expression levels. Optionally, an unchanged list may not be provided, and all genes not present in the first list will serve as the background set. Instead of a gene list the user may provide a list of genes with associated fold change values (or any other metric used in high-throughput experiments) be provided instead. In the latter case the changed and unchanged gene lists are produced by sorting all genes according to the metric provided and using a user-defined number of genes as “changed”. Optionally, the user may use a miRNA filter, using a list of miRNAs of interest to calculate results only for hexamers corresponding to these miRNAs. This option simplifies the results page, and is especially useful when a miRNA expression measurement has been performed along the gene expression experiment.

### AU Normalization on Microarray Data

**Figure 3:**
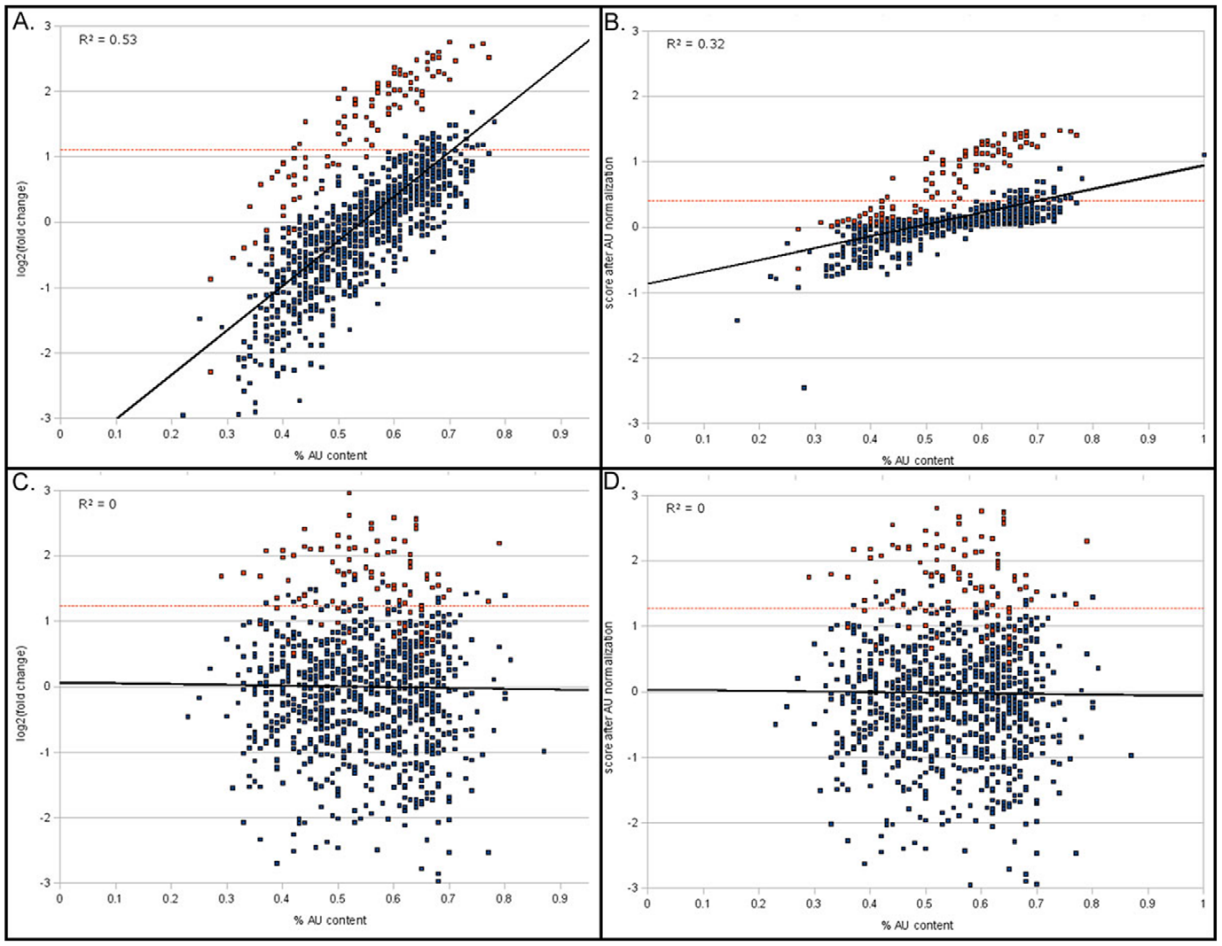
Results of AU correction. For 1000 genes, out of which 100 are upregulated (red points) and 900 are stable (blue points) the log2(fold change) is plotted against the percentage of As or Us in the 3′UTRs of genes. The top panels (A,B) show data with a linear AU bias and the bottom panels (C,D) show data with no AU bias. The left panels (A,C) show original data and the right panels (B,D) show data after AU correction. An optimal linear fit (black line) passes through the data with a correlation coefficient (R^2^) denoted for each panel. A dotted red line denotes the 100 genes with the highest log2(fold change) values.

When the input data is provided as microarray fold change levels, a single nucleotide composition bias may arise [10]. Single nucleotide AU normalization has been shown to improve the identification of miRNA signatures from microarray data. DIANA-mirExTra optionally provides such normalization as shown in Figure 3. When a bias is present the AU normalization option will diminish the correlation between AU composition and gene expression changes (Figure 3a, 3b). Moreover, when a bias is not present, the AU correction step will not significantly affect input values (Figure 3c, 3d).

### Wilcoxon Test

**Figure 4:**
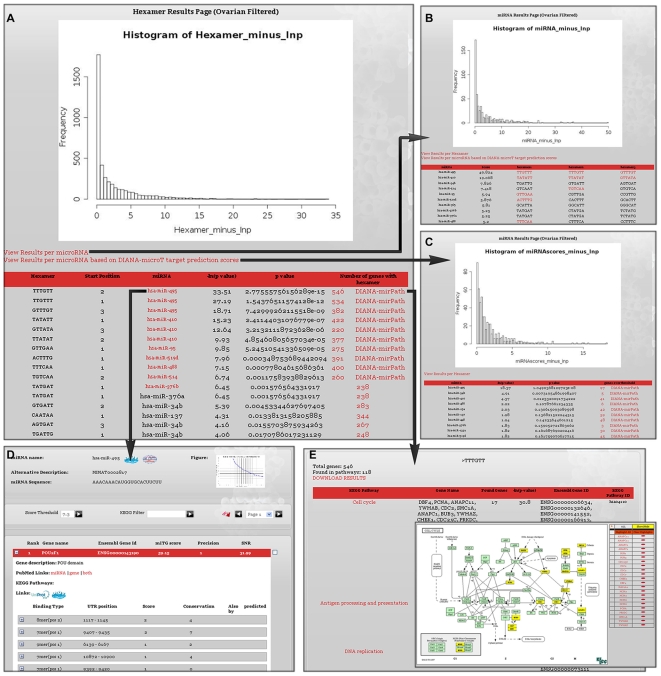
Results Page and links (Epithelial Ovarian Cancer). Genes upregulated and miRNAs downregulated in late stage Epithelial Ovarian Cancer (EOC) compared to early stage EOC were run through DIANA-mirExTra. The main results page (A) consists of two parts. At the top of the page is the histogram of the distribution of –lnp values for all possible hexamers and at the bottom, the sorted list of hexamers that can be mapped on deregulated miRNAs with corresponding p-values. The same hexamer can be shown multiple times if it can be mapped on more than one miRNAs. Hexamers are sorted according to p-value and negative natural logarithm (-lnp value). Following the link “View Results per microRNA” the user is taken to a page (B) showing miRNAs sorted according to a combinatorial score produced by the values of hexamers 1 and 2. The link “View Results per microRNA based on DIANA-microT target prediction scores” leads to a similar results page (C) that uses as a measure the scores of each gene according to miRNA target prediction program DIANA-microT. (D) Genes that contain at least one of the top ten hexamers are marked in the results page of DIANA-microT. The DIANA-microT results page for each miRNA can be found following the link on the miRNA name from the first results page. Additionally, links to DIANA-mirPath lead to a page (E) showing functional analysis results using this program. Genes containing the hexamer of interest (A), or targeted by the miRNA of interest (C) are mapped on KEGG pathways and the most significantly overrepresented pathways can be identified by their corresponding p-values.

After the input gene lists have been determined, we proceed to compare the distributions of all possible hexamers on the 3′UTR sequences between them. A one-sided Wilcoxon Rank Sum test is used in order to identify hexamers that are present significantly more often in the set of changed genes compared to the background of unchanged genes, as has been previously proposed [5]. A probability value (p-value) for each motif is calculated signifying the probability that the changed and unchanged sets are produced by the same distribution and the differences between them are due to chance alone. As a more intuitive measure, the equivalent negative natural logarithm of the p-value (-lnp) is generally used. A histogram of the distribution of -lnp values of all motifs is provided in the results page so that the user may visually evaluate the significance of the results for a motif or miRNA of interest (Figure 4). Hexamers are mapped back onto the first 8 nucleotides of a miRNA (Figure 1), known to be the most important for the miRNA:mRNA binding [11], [12].

### Combination of Hexamers

**Figure 5:**
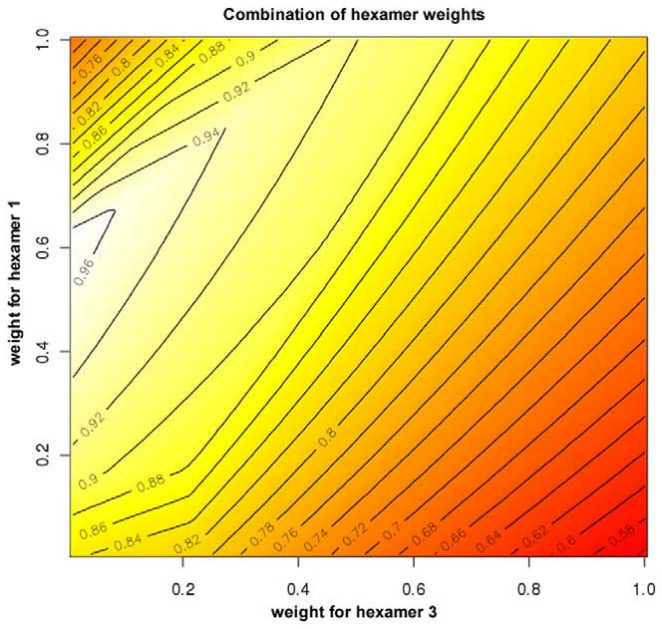
Combination of weighted -lnp values of the three hexamers. The weight for hexamer 1 is on the Y axis, for hexamer 2 is held constant at a value of 1, and for hexamer 3 is on the X axis. The mean normalized difference of the correct miRNA versus the next highest miRNA was maximized for 5 datasets of knocked out miRNAs (see Methods). The optimal weights combination for hexamers 1 and 3 were identified as 0.6 and 0 respectively. The value for hexamer 3 is still given in the Results page (see Figure 4) although it is not used for the combined score calculation.

The hexamer starting at position 2 of the miRNA, frequently called the ‘seed’ hexamer (Figure 1), can be used for an approximate identification of miRNA binding sites, with identification precision similar to some dedicated target prediction algorithms (Selbach et al. 2008). However, more than one miRNAs may share the same seed hexamer. We investigated whether it is possible to distinguish between similar miRNAs by using the p-values of flanking hexamers 1 and 3. Weighted -lnp values of three hexamers corresponding to each miRNA were summed using different weights to produce a total hexamer score (Figure 5). As a result, DIANA-mirExTra provides a combinatorial hexamer score in which the -lnp value of hexamer 1 is multiplied by a weight of 0.6 and added to the -lnp value of hexamer 2, and hexamer 3 is not taken This approach allows a single score per miRNA that takes into account the whole active region of the 8 first nucleotides of the miRNA.

### Conserved Hexamers

Hexamers corresponding to miRNAs represent an extremely loose definition of miRNA target sites. Arguably most of the hexamers present on the 3′UTR of a gene will not be parts of active miRNA target sites. Interspecies conservation has been extensively used by miRNA target prediction programs in order to refine predictions of putative miRNA target sites. Conservation of hexamers between human and mouse sequences can be optionally used in DIANA-mirExTra for a stricter and more precise definition of miRNA target sites. This option prevents a part of randomly occurring hexamers from being counted as miRNA targets, but will be intrinsically biased towards miRNAs strongly conserved between the human and murine genomes.

### Use of Target Prediction

Another option provided by DIANA-mirExTra is the use of miRNA target prediction scores instead of hexamer frequencies on a 3′UTR. A one-sided Wilcoxon Rank Sum test is performed for each miRNA, between the target prediction scores of the list of ‘changed’ genes versus the target prediction scores of the list of ‘unchanged’ genes. Target prediction scores are calculated by DIANA-microT [13], [14], an advanced miRNA target prediction program that takes into account diverse features such as evolutionary conservation in several species and weights for different types of binding sites.

### Meta-Analysis: Integration with DIANA-mirPath

After results are produced, a link to the results page is returned to the user via email. Runs typically take approximately 10 minutes. The main DIANA-mirExTra Results Page (Figure 4a) shows p-values associated with each hexamer sorted in order of significance. A histogram of the -lnp values of all possible hexamers allows the user to evaluate the significance of the p-values of a given motif. Links to Results pages for combined motifs and target prediction score results allow the user to navigate to these pages (Figure 4b,4d). For the targets of each miRNA belonging to the set of ‘changed’ genes a link to functional analysis using DIANA-mirPath [15] is provided (Figure 4c). DIANA-mirPath is a tool that identifies KEGG pathways [16], [17] enriched in the genes of interest. Such functional analysis may help to elucidate the biological function of a miRNA implicated in the condition of interest.

### Evaluation

DIANA-mirExTra was tested on several experimental datasets in which a single miRNA has been artificially deregulated, and mRNA levels measured using microarrays. In such a high throughput experiment [4], human miRNA hsa-miR-1 was overexpressed in HeLa cells and the mRNA levels of protein coding genes were measured by microarray before and after the introduction of the miRNA. Using a set of 82 genes identified as downregulated in the original paper, we have identified the three hexamers associated with hsa-miR-1 as the most significantly overrepresented hexamers and the combined score of hsa-miR-1 as the top ranking score. In the same paper a similar experiment was performed with the overexpression of hsa-miR-124 in HeLa cells. All three hexamers corresponding to hsa-miR-124 achieved the maximum -lnp value and consequently the combined score of hsa-miR-124 was also the top-ranking one. In other experiments involving the repression of miRNA functionality using ‘antagomirs’ [5] and miR-155 deficient mice, DIANA-mirExTra has correctly identified the repressed murine miRNA in both occasions (mmu-miR-122a and mmu-miR-155) using microarray data. For both experiments the miRNA in question is found as top of the combined scores list, with a large difference in combined score to the second miRNA.

Beyond expression microarray data, DIANA-mirExTra was also tested on high-throughput protein data. In a recent set of experiments [18], a large number of proteins were identified as downregulated after overexpression of each of five miRNAs (let-7b, miR-155, miR-16, miR-1, miR-30a) and pulsed stable isotope labeling with amino acids in cell culture (pSILAC) assays. DIANA-mirExTra was used to identify the implicated miRNA in each of these cases. The hexamer in position 2 has been found as the top ranking hexamer with the maximum possible -lnp value in all datasets. All results pages for datasets mentioned above can be openly accessed online at http://diana.cslab.ece.ntua.gr/hexamers/prec_results.php.

An early version of DIANA-mirExTra has been used in order to identify multiple miRNAs involved in the progression from early to late stage Epithelial Ovarian Cancer (EOC) [19]. Among other experiments, 76 EOC specimens (8 early and 68 late stage EOC) were analyzed using microarrays and 948 genes were identified as significantly upregulated in late stage EOC. A further 15212 genes were considered as unchanged between the two cancer stages. Using this data, the DIANA-mirExTra algorithm was effectively used to predict twelve miRNAs as significant candidates possibly contributing to late-stage EOC. Five of these twelve miRNAs were located on a specific miRNA gene cluster (*Dlk1 – Gtl2* domain on chr14) suggesting that this miRNA cluster could possibly be involved with EOC progression to the late stage. Further experiments showed that the miRNA gene cluster identified by DIANA-mirExTra is commonly altered in EOC and possibly other human epithelial tumors, thus validating the involvement of these miRNAs in EOC progression. Additionally, a link was established between down-regulation of the expression of miRNAs encoded in the *Dlk1 – Gtl2* cluster and higher tumor proliferation leading to shorter patient survival times. The functional analysis of predicted target genes for the top microRNAs responsible for the transition identified the “Cell Cycle”pathway as significantly related with these genes. Other cancer related pathways were strongly related to the sets of genes suggesting ways in which miRNAs may affect EOC.

## Discussion

The identification of miRNAs affecting the deregulation of genes is the primary objective of DIANA-mirExTra. Once miRNAs of interest are identified, the user can directly view predicted targets for these miRNAs as produced by DIANA-microT 3.0 [13], [14]. However, the way in which this deregulation may contribute to disease development or other processes of interest can be elucidated through functional analysis of the results. DIANA-mirExTra moves towards this direction through its direct integration with a functional analysis tool, DIANA-mirPath, suggesting biological pathways in which targets of a miRNA of interest are more probable to be involved.

With our implementation of the algorithms proposed here in a user-friendly web server we strive to allow users without expertise in data analysis to use our algorithms easily and effectively. In other relevant available software packages, that first need to be downloaded and installed locally, 3′UTR and miRNA sequences have to be provided by the user in a program-specific format. In DIANA-mirExTra sequences are automatically downloaded by the Ensembl database [20] and linked to several widely used nomenclatures. This allows the direct use of the program without the prior download of bulky sequence files and without the need to process such files to fit a predetermined format. Additionally, the program is run in a web browser, without the need for download and compilation of source code. Results are stored in an online server and are accessible from anywhere and at all times. All submitted jobs are run remotely on a dedicated computational cluster, and allow users with low computational power to use the program without experiencing long running times or memory problems

Using the simplest hexamers, the user opts for a loose definition of a miRNA target gene and may be able to identify processes not deeply conserved in other species. The option to use hexamers conserved between human and mouse provides a refinement of results for processes and miRNAs that are conserved between the two species. The stricter approach of using predicted microRNA targets as motifs takes into account conservation in several species as well as miRNA specific characteristics and could be biased towards more deeply conserved miRNAs.

Given the important role that miRNA regulation plays in several cell processes, a routine check of miRNA involvement should be encouraged even if there is no reason for it to be suspected. A simple and intuitive online tool such as DIANA-mirExTra is the obvious choice for such routine checks as it does not need complicated installation, processing of external datasets or high computational power on the user end.

## Materials and Methods

### MicroRNA and 3′UTR Sequences

MicroRNA sequences used in all predictions for DIANA-microT [13], [14] are taken from miRBase Build 10.0 [21]. 3′UTR sequences used are the longest annotated 3′UTRs from Ensembl 48 [20]. Name conversions to Ensemble gene names are done based on alternative names provided from Ensembl 48. Multiple genome alignments are downloaded from UCSC Genome Browser [22]. Human (hg18) alignment to 16 vertebrate genomes and Mouse (mm9) alignment to 29 vertebrate genomes are used.

### Hexamers

Non-overlapping six nucleotide long motifs (hexamers) are counted on the 3′UTR sequence of protein coding genes provided by Ensembl. The count of hexamers is divided by the length of the 3′UTR sequence to calculate normalized counts (hexamers/nt).

### Combination of Hexamers

The difference between the score of the ‘correct’ miRNA and the next best miRNA that did not have all three same hexamers was calculated and divided by the score of the ‘correct’ miRNA. The sum of these differences for five protein data sets [18] was maximized. The sum was calculated for all combinations of weights for hexamer 1 and 3 in 0.01 intervals for values between 0 and 1 (Figure 5). Keeping the weight for the ‘seed’ hexamer constant at 1, we have determined that for a weight of hexamer 1 set to 0.6, no value of hexamer 3 will improve the identification of the correct miRNA. Therefore DIANA-mirExTra provides a combinatorial hexamer score in which the -lnp value of hexamer 1 is multiplied by a weight of 0.6 and added to the -lnp value of hexamer 2. Hexamer 3 is not taken into account for the calculation of the combinatorial hexamer score.

### Conservation

There is the option to use only hexamers perfectly conserved on the 3′UTRs of human and mouse based on multiple species alignments downloaded from UCSC Genome Browser.

### Wilcoxon Rank Sum Test

The statistical package R is used to perform the Wilcoxon Rank Sum Test between counts or scores of ‘changed’ and ‘unchanged’ genes. The function wilcox.exact(exactranktests) is used for the one-sided test. The maximum p-value that this method may produce is 10^−19^ which is equal to -lnp = 43.74

### AU Bias Correction

When microarray data with fold change values are used as input, an optional AU content intensity bias removal step is allowed as described by Elkon and Agami [10]. The statistical package R is used for the correction, and specifically the scatter plot smoothing function lowess using default parameters. Artificial data plotted in Figure 3 consists of 1000 values with a linear correlation to AU composition (Figure 3a, Figure 3b) or no correlation to AU composition (Figure 3c, Figure 3d). The difference of the means between the 100 “upregulated” genes (red spots) and the 900 “unchanged” genes (blue spots) is the same between Figure 3a and Figure 3c. Normally distributed noise has been added to both sets. Several other artificially produced examples with varying differences and levels of AU bias were produced (data not shown) with similar results.
